# Rapid defense responses in maize leaves induced by *Spodoptera exigua* caterpillar feeding

**DOI:** 10.1093/jxb/erx274

**Published:** 2017-08-22

**Authors:** Vered Tzin, Yuko Hojo, Susan R Strickler, Lee J Bartsch, Cairo M Archer, Kevin R Ahern, Shaoqun Zhou, Shawn A Christensen, Ivan Galis, Lukas A Mueller, Georg Jander

**Affiliations:** 1 Boyce Thompson Institute for Plant Research, Tower Rd, Ithaca, NY, USA; 2 Okayama University, Institute of Plant Science and Resources, Kurashiki, Okayama, Japan; 3 USDA-ARS Chemistry Unit, Center for Medical, Agricultural, and Veterinary Entomology, Gainesville, FL, USA

**Keywords:** Benzoxazinoid, insect herbivore, jasmonic acid, metabolite profile, RNAseq, *Spodoptera exigua*, time course, transcriptome, *Zea mays*

## Abstract

Insects such as the beet armyworm (*Spodoptera exigua*) cause extensive damage to maize (*Zea mays*). Maize plants respond by triggering defense signaling, changes in gene expression, and biosynthesis of specialized metabolites. Leaves of maize inbred line B73, which has an available genome sequence, were infested with *S. exigua* for 1 to 24 h, followed by comparisons of the transcript and metabolite profiles with those of uninfested controls. The most extensive gene expression responses occurred rapidly, within 4–6 h after caterpillar infestation. However, both gene expression and metabolite profiles were altered within 1 h and continued to change during the entire 24 h experiment. The defensive functions of three caterpillar-induced genes were examined using available *Dissociation* transposon insertions in maize inbred line W22. Whereas mutations in the benzoxazinoid biosynthesis pathway (*Bx1* and *Bx2*) significantly improved caterpillar growth, the knockout of a 13-lipoxygenase (*Lox8*) involved in jasmonic acid biosynthesis did not. Interestingly, 9-lipoxygenases, which lead to the production of maize death acids, were more strongly induced by caterpillar feeding than 13-lipoxygenases, suggesting an as yet unknown function in maize defense against herbivory. Together, these results provide a comprehensive view of the dynamic transcriptomic and metabolomic responses of maize leaves to caterpillar feeding.

## Teaser

A comprehensive transcriptomic and metabolomic profiling time course of maize foliar responses to caterpillar feeding identified dynamic changes in the expression of genes related to the synthesis of benzoxazinoids and phytohormones.

## Introduction

Plants perceive herbivory through mechanical cues from feeding damage, oviposition, and even insects walking on the leaf surface (Mithöfer *et al.*, 2005; Hilker and Meiners, 2006), as well as through chemical cues from insect oral secretions and frass (Alborn *et al.*, 1997; Ray *et al.*, 2015). Perception of insect feeding leads to the induced production of physical and chemical defensive mechanisms that promote plant fitness (Zhou *et al.*, 2015), as well as a reduction of major cell processes involved in growth and photosynthesis (Appel *et al.*, 2014). Several phytohormones function in regulating plant defense, including jasmonic acid (JA), salicylic acid (SA), abscisic acid (ABA), ethylene, auxin, and cytokinins (Staswick *et al.*, 2002; Koornneef and Pieterse, 2008; Frébortová *et al.*, 2010; Zeier, 2013; Westfall *et al.*, 2016; Urano *et al.*, 2017). However, JA and SA and their derivatives play a predominant role in modulating plant defense against pests and pathogens, respectively (Howe and Jander, 2008; Wu and Baldwin, 2010). Jasmonates, in particular, regulate the production of toxic metabolites and a wide variety of other responses to insect herbivory (Kessler *et al.*, 2004; Schmelz *et al.*, 2011; Christensen *et al.*, 2013).

**Fig. 1.**
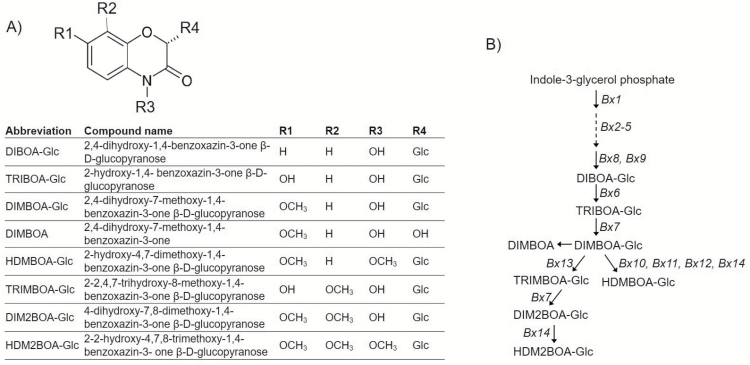
Benzoxazinoid biosynthesis in maize. (A) Structures of maize benzoxazinoids. (B) The benzoxazinoid biosynthesis pathway in maize. Known enzymes and key pathway metabolites are indicated.

In some graminaceous plants, including maize (*Zea mays*), wheat (*Triticum aestivum*), and rye (*Secale cereale*), JA induces the production of benzoxazinoids (Fig. 1A), a class of metabolites that can provide protection against insect herbivores, pathogens, and competing plants (Oikawa *et al.*, 2001, 2004; Frey *et al.*, 2009; Adhikari *et al.*, 2015; Wouters *et al.*, 2016). In maize, a series of nine enzymes (Bx1–Bx9) catalyse the biosynthesis of 2,4-dihydroxy-7-methoxy-1,4-benzoxazin-3-one glucoside (DIMBOA-Glc) from indole-3-glycerol phosphate (Frey *et al.*, 1997; Niemeyer, 2009) (Fig. 1B). The first committed step of the benzoxazinoid pathway is conversion of indole-3-glycerolphosphate to indole by Bx1, followed by conversion of indole to DIBOA through oxidation at four positions by cytochrome P450-dependent monooxygenases, Bx2–Bx5 (Frey *et al.*, 2009). *Dissociation* (*Ds*) transposon mutations knocking out the first two genes in the pathway, *Bx1* and *Bx2*, lead to improved growth of corn leaf aphids (*Rhopalosiphum maidis*) (Tzin *et al.*, 2015*a*). A family of three *O*-methyltransferases (Bx10–Bx12) methylates DIMBOA-Glc to form 2-hydroxy-4,7-dimethoxy-1,4-benzoxazin-3-one glucoside (HDMBOA-Glc) (Meihls *et al.*, 2013). DIMBOA-Glc and HDMBOA-Glc are the most prevalent benzoxazinoids in maize seedlings (Frey *et al.*, 1997, 2009), though their relative abundance is quite variable among different maize inbred lines (Meihls *et al.*, 2013). Recently, two additional enzymatic steps in this pathway were identified: first, a 2-oxoglutarate-dependent dioxygenase (Bx13) that catalyses the conversion of DIMBOA-Glc into 2,4,7-trihydroxy-8-methoxy-1,4-benzoxazin-3-one glucoside (TRIMBOA-Glc) and second, an *O*-methyltransferase (Bx14) that converts 2,4-dihydroxy-7,8-dimethoxy-1,4-benzoxazin-3-one glucoside (DIM2BOA-Glc) into 2-hydroxy-4,7,8-trimethoxy-1,4-benzoxazin-3-one glucoside (HDM2BOA-Glc) (Handrick *et al.*, 2016) (Fig. 1). Feeding by chewing herbivores brings benzoxazinoid glucosides into contact with β-glucosidases, leading to the formation of toxic breakdown products (Esen, 1992; Oikawa *et al.*, 1999; Niemeyer, 2009). Induced benzoxazinoid accumulation and methylation of DIMBOA-Glc to produce HDMBOA-Glc in response to caterpillar feeding (Oikawa *et al.*, 2004; Tzin *et al.*, 2015*b*) has been associated with increased resistance to several lepidopteran herbivores, including *Spodoptera exigua* (beet armyworm), *Spodoptera littoralis* (Egyptian cotton leafworm), *Spodoptera frugiperda* (fall armyworm), and *Diatraea grandiosella* (southwestern corn borer) (Hedin *et al.*, 1993; Oikawa *et al.*, 2001; Niemeyer, 2009; Glauser *et al.*, 2011).

In the present study, we investigated the dynamic maize responses to *S. exigua* caterpillar feeding by integrating results from high-throughput RNA sequencing with phytohormone and metabolite profiling experiments. Our study focused on the generalist lepidopteran herbivore *S. exigua*, which is a serious pest of grains, vegetables, flower crops, and occasionally trees (Kim and Kim, 1997). Our experiments involved two well-characterized maize inbred lines: B73 for transcriptomic analysis because of the sequenced genome (Schnable *et al.*, 2009) and W22 for knockout mutations because of the available *Ds* transposon insertion mutations (Vollbrecht *et al.*, 2010). Leaves of the maize inbred line B73 were infested with *S. exigua* caterpillars for 1, 4, 6, or 24 h, and statistical analyses were conducted to identify patterns of gene expression and metabolite changes.

## Materials and methods

### Plants and growth conditions

Single maize seeds were planted in 7.6 × 7.6-cm plastic pots (200 cm^3^), 1.5 cm deep, filled with moistened maize mix, which was produced by combining 0.16 m^3^ Metro-Mix 360 (Scotts, Marysville, OH, USA), 0.45 kg finely ground lime, 0.45 kg Peters Unimix (Griffin Greenhouse Supplies, Auburn, NY, USA), 68 kg Turface MVP (Banfield-Baker Corp., Horseheads, NY, USA), 23 kg coarse quartz sand, and 0.018 m^3^ pasteurized field soil. Plants were grown for 2 weeks in growth chambers under a controlled photoperiod regime with a 16 h light/8 h dark cycle, 180 mmol photons m^−2^ s^−1^ light intensity at constant 23 °C and 60% humidity.

### Caterpillar bioassays

*Spodoptera exigua* eggs were purchased from Benzon Research (Carlisle, PA, USA). After incubation for 48 h in a 29 °C incubator, first instar caterpillars were transferred to an artificial diet (Beet Armyworm Diet, Southland Products Inc., Lake Village, AR, USA). Control and experimental maize seedlings received clip cages on the third leaf for 24 h. For measuring the effect of caterpillar feeding on the maize transcriptome and metabolome, second to third instar *S. exigua* caterpillars were added to the clip cages for the final 1, 4, 6, or 24 h of the experiment (Supplementary Fig. S1 at *JXB* online). All plant material was harvested at the same time. For *bx1::Ds* and *bx2::Ds* maize seedling caterpillar bioassays, individual caterpillars were confined on 10-day-old plants with micro-perforated polypropylene bags (15 cm×61 cm; PJP Marketplace, http://www. pjpmarketplace.com), and caterpillar fresh weight was measured 4 d after the start of infestation.

### Total RNA extraction

Leaf material was harvested, flash-frozen in liquid nitrogen, and ground to a fine powder using a paint shaker (Harbil, Wheeling, IL, USA) and 3-mm-diameter steel balls (Abbott Ball, West Hartford, CT, USA). After sample homogenization, RNA was extracted using TRI Reagent (Sigma-Aldrich, St Louis, MO, USA) and purified with the SV Total RNA isolation kit with on-column DNase treatment (Promega, Madison, WI, USA). Total RNA concentration and quality were assessed using a NanoDrop instrument (2000c; Thermo Fisher Scientific Inc., Waltham, MA, USA).

### Transcriptome sequencing, RNAseq data analysis and qRT-PCR analysis

Tissue from three individual maize plants was combined into one experimental replicate, and four replicates were collected for each time point. The purified total RNA (2–3 μg) was used for the preparation of strand-specific RNAseq libraries (Zhong *et al.*, 2011; Chen *et al.*, 2012) and amplified for 16 cycles. The purified RNAseq libraries were quantified, and 20 ng of each was used for next-generation sequencing using an Illumina HiSeq2000 instrument (Illumina, San Diego, CA, USA) at Weill Cornell Medical School (New York, NY, USA) with a 101 bp single-end read length. Libraries were multiplexed and sequenced in one lane. Read quality values were checked using FastQC (http://www.bioinformatics.babraham.ac.uk/projects/fastqc). Low-quality sequences and adapters were trimmed and removed using Fastq-mcf (https://github.com/ExpressionAnalysis/ea-utils/blob/wiki/FastqMcf.md), with a minimum length of 50 bp and a minimum quality value of 30. RNAseq analysis was performed following a previously published protocol (Anders *et al.*, 2013), using the maize genome version B73 AGP v3.22 as a reference (Zhong *et al.*, 2011). The benzoxazinoid genes were also analysed using AGP v3.20 to determine the expression levels of *Bx7* and *Bx13*, which were excluded from the AGP v3.22 as low-confidence genes. Reads were mapped with TopHat2 (Kim *et al.*, 2013) followed by expression analysis using the Cuffdiff package (Trapnell *et al.*, 2012) version 2.2.1, using the geometric mean option. Transcripts showing at least one fragment per kilobase of exon per million fragments (FPKM) of transcript in three or more replicates for each time point were kept for differentially expressed gene detection. To verify the results of the RNA-seq analysis, an additional experiment was conducted, and total RNA was extracted. First-strand cDNA was synthesized by M-MLV reverse transcriptase (TaKaRa Bio USA, Mountain View, CA, USA), and the library was used as templates for qRT-PCR analysis, as described previously (Tzin *et al.*, 2015*a*). The primer sets used to amplify the seven genes are given in Supplementary Table S1.

### Targeted and untargeted metabolite assays

For assays of maize metabolites, approximately 2 cm of caterpillar-fed third leaf tissue was collected in parallel with control leaves without caterpillars. For non-targeted metabolite assays, frozen powder ground from fresh tissue was weighed in a 1.5 ml microcentrifuge tube, and extraction solvent (methanol/water/formic acid, 70:29.9:0.1, v/v/v) in a 1:3 ratio was added to each sample (Mijares *et al.*, 2013). The tubes were briefly vortexed, shaken for 40 min at 4 °C, and centrifuged for 5 min at 14 000 *g.* The samples were filtered through a 0.45 μm filter plate (EMD Millipore Corp., Billerica, MA, USA) by centrifuging at 2000 *g* for 3 min; then the supernatant was diluted 1:9, and the extraction solvent subsequently transferred to an HPLC vial. Liquid chromatography–tandem mass spectrometry (LC-MS/MS) analysis was performed on a Dionex UltiMate 3000 Rapid Separation LC System attached to a 3000 Ultimate diode array detector and a Thermo Q-Exactive mass spectrometer (Thermo Fisher Scientific). The samples were separated on a Titan C18 7.5 cm×2.1 mm×1.9 μm Supelco Analytical Column (Sigma-Aldrich), as previously described (Handrick *et al.*, 2016). Raw mass spectrometry data files were converted using XCMS (Smith *et al.*, 2006), followed by data analysis using the CAMERA R package (Kuhl *et al.*, 2012). The chromatographic peaks were compared with the retention time, accurate mass and UV spectrum of standards of DIMBOA, DIMBOA-Glc, and HDMBOA-Glc. Other benzoxazinoids were identified based on their accurate masses and UV spectra. Benzoxazinoid levels and identification were calculated from standard curves that were produced with authentic standards provided by Gaetan Glauser (Supplementary Table S2; University of Neuchatel, Neuchatel, Switzerland).

### Phytohormone analysis

Maize leaves (30–100 mg fresh weight) were harvested, frozen in liquid nitrogen, and lyophilized. The analysis was performed as described previously (Fukumoto *et al.*, 2013), with some modifications in sample cleanup before LC-MS/MS analysis. Samples were homogenized in a FastPrep®-24 (MP Biochemicals, Santa Ana, CA, USA) using five 2.3-mm-diameter zirconia beads and 1 ml of ethyl acetate solvent spiked with deuterated internal standards (25 ng d3-JA, 5 ng d3-JA-Ile, 10 ng d6-abscisic acid (ABA), and 20 ng d4-SA). Samples were centrifuged for 15 min at 13 200 *g*, 4 °C, and supernatants were collected in clean 2 ml microcentrifuge tubes. Pellet extraction was repeated once with 0.5 ml of pure ethyl acetate and vortexing for 5 min at 23 °C; supernatants were pooled with the previous fraction after centrifugation. Samples were dried completely under a vacuum in a miVac Quatro concentrator (Genevac Ltd, Ipswich, UK). Each sample was dissolved in 300 µl of 70% methanol/water (v/v) and vortexed for 5 min at 23 °C. Then, 1700 µl of buffer (84 mM ammonium acetate; pH 4.8) was added to each sample prior to application and retention of phytohormones on preconditioned 3 ml SPE columns (Bond Elut-C18, 200 mg, Agilent Technologies Inc., Santa Clara, CA, USA) set in a QIAvac 24 Plus system (Qiagen, Germantown, MD, USA). After a brief drying period with an air stream, samples were eluted with 800 µl of 85% methanol/water (v/v) into clean 1.5 ml microcentrifuge tubes. After spinning at 12 000 *g* to remove insoluble material, 10 µl aliquots were analysed using a triple quadrupole LC-MS/MS 6410 system (Agilent Technologies) equipped with a Zorbax SB-C18 column [2.1 mm i.d.×50 mm (1.8 µm), Agilent Technologies] kept in a thermostat-controlled chamber at 35 °C. The solvent gradient, A (0.1% formic acid in water) *vs* B (0.1% formic acid in acetonitrile), was used as follows: 0 min, 15% B; 4.5 min, 98% B; 12 min 98% B; 12.1 min, 15% B; and 18 min,15% B, at a constant flow rate of 0.4 ml min^−1^. Mass transitions, hormone/Q1 precursor ion (*m*/*z*)/Q3 product ion (*m*/*z*), were monitored for each compound as follows: JA/209/59, JA-Ile/322/130, ABA/263/153, SA/137/93, 12-oxo-phytodienoic acid (OPDA)/291/165, hydroxy (OH)-JA/225/59, OH-JA-Ile/338/130, carboxy (COOH)-JA-Ile/352/130, JA-Val/308/116, d3-JA/212/59, d3-JA-Ile/325/130, d6-ABA/269/159, and d4-SA/141/97. The fragmentor (V)/collision energy (V) parameters were set to 100/6 for JA, OH-JA, and OPDA; 135/15 for JA-Ile, OH-JA-Ile, COOH-JA-Ile, and JA-Val; 130/5 for ABA; and 90/12 for SA. The JA, JA-Ile, ABA, and SA amounts were directly calculated from the ratio of the endogenous hormone peak and the known deuterated internal standard. Compounds for which the authentic deuterated standards were not available were quantified using their structurally nearest deuterated internal standard, and expressed as equivalents of this compound (OPDA as d3-JA eq.; OH-JA-Ile, COOH-JA-Ile, and JA-Val as d3-JA-Ile eq.). Phytohormone concentrations were calculated relative to the actual fresh mass of each sample used for extraction (Supplementary Table S3).

### Isolation of transposon insertion knockout lines and genotyping

The *lox8/ts1-ref* mutant was acquired from the Maize Genetics Cooperation Stock Center at the University of Illinois at Urbana-Champaign (Maize COOP, http://maizecoop.cropsci.uiuc.edu) as a segregating 1:1 heterozygous:mutant population. Heterozygous plants were identified and self-pollinated to generate a 1:2:1 segregating population, as described previously (Christensen *et al.*, 2013). *Ds* transposon insertions in the W22 inbred background were identified in our genes of interest through the *Ac*/*Ds* tagging project website (http://www.acdstagging.org) (Vollbrecht *et al.*, 2010). Seed stocks were acquired for *bx1::Ds* (Gene ID: GRMZM2G085381; Ds: B.W06.0775), *bx2::Ds* (Gene ID: GRMZM2G085661; Ds: I.S07.3472) and *lox8(ts1)::Ds* (Gene ID: GRMZM2G104843; Ds: B.S06.0143). The Maize Genetics Cooperation Stock Center seed stock ID numbers and the primer sets used to confirm the mutant lines are given in Supplementary Table S4.

### Statistical analysis

Principal component analysis (PCA) was conducted and plotted using MetaboAnalyst 3.0 software (Xia *et al.*, 2009). Venn diagrams were made using the Venny 2.1.0 drawing tool (http://bioinfogp.cnb.csic.es/tools/venny/index.html). The optimal number of clusters for the transcriptomic data used for the *k*-means clustering analysis was calculated using the Gap (Tibshirani *et al.*, 2001) and NbClust R packages (Charrad *et al.*, 2014). The *k*-means analysis was performed on scaled and centered FPKM log2 values and presented in a standard *Z*-score format. A gene ontology enrichment analysis was conducted using the PlantGSEA tool (http://structuralbiology.cau.edu.cn/PlantGSEA/) (Yi *et al.*, 2013). Fisher’s exact test was used to take into account the number of genes in the group query, the total number of genes in a gene set, and the number of overlapping genes, with the false discovery rate calculated using the Hochberg procedure (*P* value=0.05). Statistical comparisons were made using JMP Pro 11 (www.jmp.com, SAS Institute, Cary, NC, USA).

## Results and discussion

### Transcriptomic analysis of maize responses to caterpillar feeding

To investigate global transcriptomic changes in response to caterpillar feeding, the third leaves of maize inbred line B73 seedlings were infested with two second–third instar *S. exigua* caterpillars for 1, 4, 6, and 24 h. A recent investigation of the effects of *S. littoralis* feeding on maize defense mechanisms showed that induced herbivore resistance is highly localized and dependent on benzoxazinoid biosynthesis (Maag *et al.*, 2016). Therefore, we focused our transcriptomic assays on the caterpillar-infested section of the leaf. Caterpillar exposure was initiated in a staggered manner, such that all samples were harvested in the early afternoon at the same time on the same day (Supplementary Fig. S1). A comparison of transcriptome data (Illumina RNAseq) with annotated gene models found in the B73 reference genome sequence (AGPv3.22; www.maizegdb.org) (Zhong *et al.*, 2011) revealed approximately 40 000 transcripts (Supplementary Table S5). The expression patterns of six selected genes, relative to a housekeeping gene (Adenine Phosphate Transferase 1; GRMZM2G131907), were confirmed by quantitative reverse transcription-PCR (qRT-PCR) using an independently generated cDNA library. A comparison of gene expression using these two methods showed a similar expression pattern and a high correlation coefficient (*R*-value; Supplementary Fig. S2). We excluded genes with low expression values by filtering out all those that had no detectable expression in at least three samples in the data set. This process yielded approximately 20 000 transcripts from the RNAseq dataset that were analysed for each of the four caterpillar-infested time points to detect genes that were differentially expressed relative to untreated control leaves (Supplementary Table S6).

**Fig. 2.**
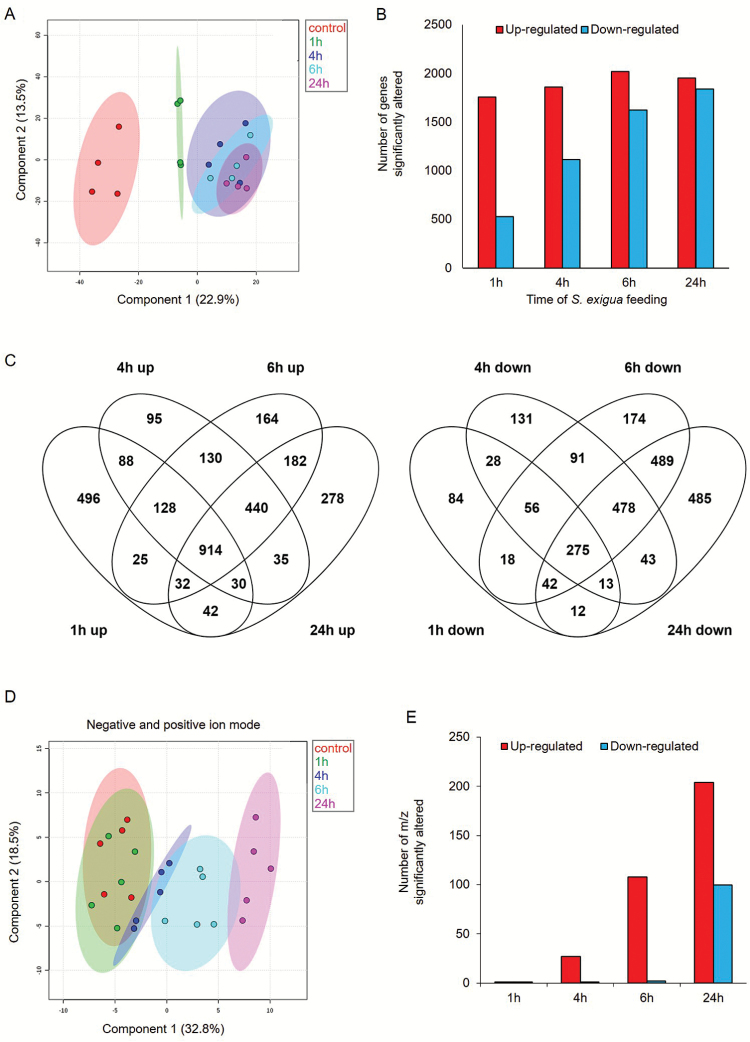
Transcriptomic and metabolomic overview of a time course of *S. exigua* feeding on maize inbred line B73 foliage. (A) PCA plot generated using 20 825 genes (FPKM>0 in at least 18 samples). Ovals indicate 95% confidence intervals. (B) Total number of transcripts that were significantly up- or down-regulated. (C) Venn diagram illustrating the number of genes up- or down-regulated by caterpillar infestation in the time course. *P*<0.05 FDR and fold change >2 or <0.5. (D, E) Untargeted metabolomics of maize leaf responses to caterpillar feeding. (D) PCA plot of negative (1024 electrospray ionization; ESI) and positive (1274 ESI) mass signals, filtered using Metaboanalyst software. Ovals indicate 95% confidence intervals. (E) Total number of mass signals that were significantly up- or down-regulated. (This figure is available in color at *JXB* online.)

The gene expression levels were used to conduct a PCA for each of the biological replicates (Fig. 2A). Samples from each time point clustered with one another, and the expression profiles gradually separated over time from the 0 h (control) sample. Samples from the 24-h time point clustered furthest from the control samples, indicating that the greatest changes in gene expression occurred after the onset of caterpillar feeding. Genes with significant expression differences (*P*≤0.05, false-discovery rate (FDR) adjusted) and at least two-fold changes relative to the controls for at least one of the time points were selected for further analysis (Supplementary Table S7). After the initiation of caterpillar feeding, thousands of transcripts showed altered expression at each of the time points. The number of down-regulated genes increased gradually over the time course, culminating in similar numbers of up- and down-regulated genes in the 24-h sample (1838 down-regulated and 1954 up-regulated; Fig. 2B). The distribution of up- and down-regulated genes was calculated for each time point and is presented in a Venn diagram (Fig. 2C). Although a unique set of genes increased at each time point (total 3078), the expression of a large number of genes (914) was induced at all time points. In addition, a unique set of genes decreased at each time point (total 2419), and only 275 genes decreased at all four time points. A comparison of genes altered by caterpillar feeding after 1 and 24 h is presented in Supplementary Fig. S3.

Metabolic changes were studied in caterpillar-infested maize leaf samples using non-targeted LC-MS/MS in both the negative ion mode (Supplementary Table S8) and the positive ion mode (Supplementary Table S9). The PCA clustering pattern of the metabolite analysis is presented in Fig. 2D. The plot showed significant separation from the controls at 6 and 24 h after initiation of caterpillar feeding.

Metabolite abundance (Fig. 2E) increased more slowly over time than gene expression (Fig. 2B). As in the case of gene expression, down-regulation of metabolite abundance occurred more slowly than up-regulation (Fig. 2E; Supplementary Table S10). The overall similarity of the transcriptomic and metabolomic data suggested that caterpillar-induced gene expression changes led to induced changes in the metabolome at the same or later time points.

### Clustering the transcriptome dataset

Significantly differentially expressed genes were subjected to *k*-means clustering using Pearson correlation distances calculated from the FPKM value for each time point. The *k*-means analysis was performed on scaled and centered FPKM log2 values, and each cluster was represented by the *Z*-score (standard score) of gene expression from the set of genes showing similar response patterns to caterpillar herbivory. The 16 clusters were divided into four expression groups, derived from trends observed in the standard scores: (i) clusters with a strongly increasing average score (2 standard deviations (SD)); (ii) clusters with a moderately increasing average score (approximately 1 SD); (iii) clusters with a moderately decreasing average score (approximately 1 SD); and (iv) clusters with a moderately decreasing average score that deviated significantly from the population average (i.e. those with a high FPKM) (Supplementary Fig. S4). The gene distribution into the 16 clusters is presented in Supplementary Table S11.

To elucidate the biological processes that contributed to each gene expression cluster, an over-representation analysis was performed using PlantCyc output from the PlantGSEA tool (Yi *et al.*, 2013) (Table 1). The first pattern included two clusters of genes (1 and 2) that were highly induced by caterpillar feeding. Although these clusters contained a relatively small number of genes (133 and 74, respectively), many transcripts associated with plant defense and stress response pathways were overrepresented. These clusters included genes controlling the biosynthesis of phenylpropanoids, suberin, JA, monosaccharides, methionine, and *S*-adenosyl-L-methionine, as well as methionine degradation, the latter of which is essential for ethylene production. The second pattern included six clusters (3–8) of moderately increased gene expression, which included transcripts mostly associated with sucrose degradation and cellulose biosynthesis, but also genes involved in the tricarboxylic acid (TCA) cycle and the biosynthesis of phenylpropanoids, suberin, JA, β-alanine, glutamine, fatty acids, and cytokinin-*O*-glucosides. The moderately decreased gene expression pattern clusters (9–14) contained genes associated with photorespiration, nitrogen fixation, and flavonoid biosynthesis. Lastly, we identified a fourth pattern of genes having both high FPKM and moderately decreased expression. These two clusters (15 and 16) included genes that are involved in the photosynthesis light reaction, the Calvin–Benson–Bassham cycle, gluconeogenesis, and glycolysis (Table 1). The observed reduction in photosynthetic gene expression may have been the result of a temporal readjustment of photosynthetic capacity in response to biotic stress (Attaran *et al.*, 2014). RuBPCase activases (RCA; GRMZM2G162200 and GRMZM2G162282) are abundant photosynthetic proteins, which strongly down-regulated in response to *S. exigua* feeding (Supplementary Table S7). It was suggested that *Nicotiana attenuata* RCA is the regulator for the resource-based trade-off between growth and defense by redirecting JA conjugates (Mitra and Baldwin, 2014). However, this regulatory link has not yet been confirmed in maize.

**Table 1.** *Enrichment analysis of metabolic pathways grouped by* k*-means clustering*. Gene expression patterns were sorted into 16 clusters, as determined by *k*-means analysis of transcripts detected in the B73 maize inbred line after 0, 1, 4, 6 and 24 h of caterpillar feeding

	Cluster no.	No. of genes in the cluster	Description	No. of genes	FDR
Group 1: strong increasing average (2 SD)	1	133	Benzoate biosynthesis II (CoA-independent, non-β-oxidative)	3	3.3E−03
			Suberin biosynthesis	3	6.9E−03
			Phenylpropanoid biosynthesis, initial reactions	2	1.2E−02
			*trans*-Cinnamoyl-CoA biosynthesis	2	1.2E−02
			Adenine and adenosine salvage VI	2	1.2E−02
	2	74	*S*-Adenosyl-L-methionine cycle II	4	1.4E−06
			Jasmonic acid biosynthesis	5	1.5E−06
			Methionine degradation I (to homocysteine)	3	3.1E−05
			Traumatin and (*Z*)-3-hexen-1-yl acetate biosynthesis	3	3.5E−05
			Divinyl ether biosynthesis II	3	3.5E−05
			Linalool biosynthesis	3	9.7E−05
			*S*-Adenosyl-L-methionine biosynthesis	2	7.3E−04
			Ethylene biosynthesis from methionine	3	9.8E−04
			2′-Deoxymugineic acid phytosiderophore biosynthesis	2	2.7E−03
			UDP-D-xylose and UDP-D-glucuronate biosynthesis	2	4.1E−03
			Methylerythritol phosphate pathway	2	4.1E−03
			Tryptophan biosynthesis	2	6.5E−03
			Glycogen biosynthesis II (from UDP-D-glucose)	2	7.8E−03
			Methionine biosynthesis II	2	1.0E−02
			Colanic acid building blocks biosynthesis	2	1.9E−02
			Galactose degradation III	2	1.9E−02
Group 2: moderately increasing average (approximately 1 SD)	3	557	Homogalacturonan biosynthesis	5	1.0E−02
			Cellulose biosynthesis	6	4.3E−02
			Cytokinins-*O*-glucoside biosynthesis	8	4.3E−02
	4	493	Phenylpropanoid biosynthesis, initial reactions	3	1.8E−02
			*trans*-Cinnamoyl-CoA biosynthesis	3	1.8E−02
	5	357	Sucrose degradation I	4	1.7E−02
			Suberin biosynthesis	4	1.7E−02
	6	207	Jasmonic acid biosynthesis	6	5.3E−05
			Pyrimidine ribonucleosides degradation II	3	5.7E−04
			Methylerythritol phosphate pathway	3	1.1E−02
			β-Alanine biosynthesis II	3	3.3E−02
			Glutamine biosynthesis III	3	3.3E−02
	7	562	**No results**		
	8	408	Sucrose degradation III	8	6.8E−04
			Traumatin and (*Z*)-3-hexen-1-yl acetate biosynthesis	3	1.7E−02
			Alanine degradation II (to D-lactate)	3	1.7E−02
			Divinyl ether biosynthesis II	3	1.7E−02
			Sucrose degradation I	4	1.7E−02
			TCA cycle variation III (eukaryotic)	5	1.7E−02
			CDP-diacylglycerol biosynthesis II	4	1.8E−02
			CDP-diacylglycerol biosynthesis I	4	1.8E−02
			Triacylglycerol biosynthesis	4	2.5E−02
			Cyclopropane fatty acid biosynthesis	3	2.9E−02
			Cyclopropane and cyclopropene fatty acid biosynthesis	3	2.9E−02
			Glyoxylate cycle	3	4.9E−02
			Pyrimidine ribonucleotides interconversion	3	5.0E−02
Group 3: moderately decreasing average (approximately 1 SD)	9	456	**No results**		
	10	387	Stachyose biosynthesis	3	3.1E−02
	11	244	Photorespiration	5	4.4E−04
	12	399	Luteolin biosynthesis	3	1.3E−02
			Leucodelphinidin biosynthesis	4	1.3E−02
			Leucopelargonidin and leucocyanidin biosynthesis	4	1.3E−02
			Flavonol biosynthesis	3	3.2E−02
			Nitrogen fixation	2	4.5E−02
	13	500	**No results**		
	14	487	**No results**		
Group 4: moderately decreasing averages that significantly deviate from the population average (high FPKM)	15	150	Photosynthesis light reactions	4	2.5E−05
			Calvin–Benson–Bassham cycle	5	1.3E−03
			Gluconeogenesis I	4	2.4E−02
			Glycine cleavage complex	2	4.7E−02
			Glycolysis I	4	4.7E−02
	16	66	Methylerythritol phosphate pathway	2	5.2E−03
			Cyanate degradation	2	5.2E−03
			Photosynthesis light reactions	2	5.2E−03
			Calvin–Benson–Bassham cycle	3	5.2E−03
			Gluconeogenesis I	3	7.2E−03
			Rubisco shunt	2	3.8E−02

Phenylpropanoid metabolism, which generates an enormous array of specialized metabolites involved in many cell functions including defense and cell wall biosynthesis (Vogt, 2010), was overrepresented in three patterns (Table 1). Three metabolites from the early part of the phenylpropanoid pathway, coumaric acid, caffeic acid and ferulic acid, were identified in the LC-MS/MS dataset (Supplementary Fig. S5A). The levels of caffeic acid and ferulic acid were induced after 6 h, while the levels of all three detected phenylpropanoids declined after 24 h (Supplementary Fig. S5B). This may indicate that, after their initial synthesis, these phenylpropanoids were incorporated into other defensive metabolites (Table 1). Taken together, our LC-MS/MS data suggest that primary metabolic precursors are reallocated to synthesize specialized defense metabolites in response to *S. exigua* feeding on maize.

### Plant hormone-related genes and metabolites induced by *Spodoptera exigua* damage

**Fig. 3.**
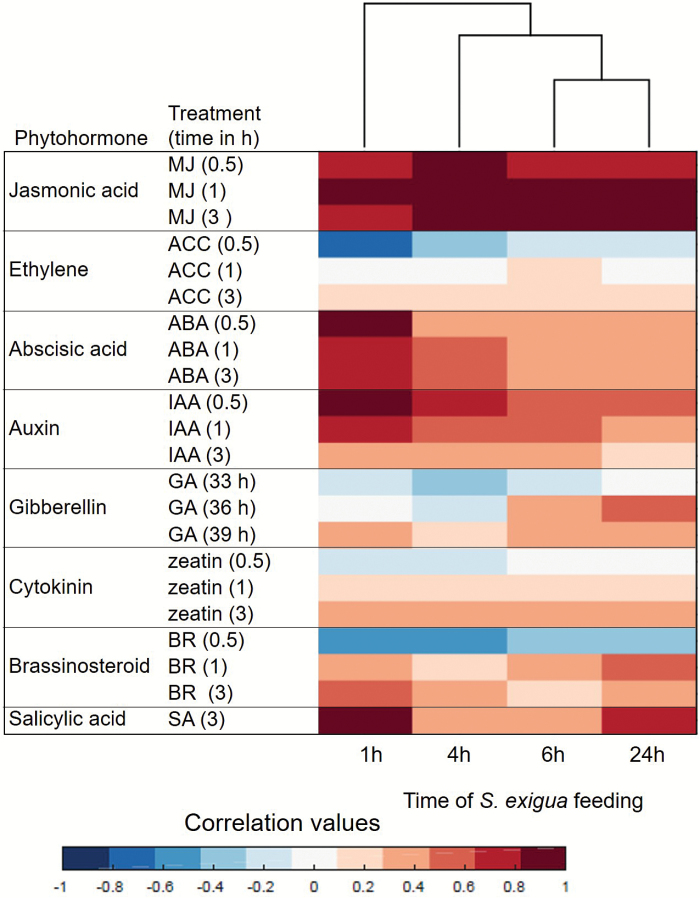
Plant hormone signatures based on transcriptomic data generated after *S. exigua* feeding on maize leaves. Red indicates a positive correlation between the maize *S. exigua* caterpillar treatment and a particular hormonal response; blue indicates a negative correlation. ABA, abscisic acid; ACC, 1-aminocyclopropane-1-caroxylic acid (precursor of ethylene); BR, brassinosteroid; GA, gibberelic acid; IAA, indole-3-acetic acid; MJ, methyl jasmonate; SA, salicylic acid. The analysis was conducted using the Hormonometer tool (Volodarsky *et al.*, 2009). (This figure is available in color at *JXB* online.)

The Hormonometer tool was used to identify the transcript signatures of maize hormonal responses to caterpillar-infested maize plants (Volodarsky *et al.*, 2009). We evaluated similarities in the expression profiles elicited by caterpillar herbivory and those induced by application of the plant hormones methyl-jasmonate, 1-aminocyclopropane-1-carboxylic acid (a precursor for ethylene), ABA, indole-3-acetic acid (auxin), cytokinin (zeatin), brassinosteroid, gibberellic acid, and salicylic acid. As the hormone treatments were conducted with *Arabidopsis thaliana* (Arabidopsis), we analysed Arabidopsis orthologs found in the B73 RefGen v.3.22 genome. Maize genes with a corresponding Arabidopsis Probeset ID were included in the filtered RNAseq analysis, which resulted in a total of 10 242 Arabidopsis orthologs used as input for the Hormonometer analysis (Supplementary Table S12). As shown in Fig. 3, genes associated with JA-, ABA-, auxin-, and SA-dependent signaling were highly induced after 1 h of infestation, followed by moderate induction of these phytohormone signaling pathways at later time points. Ethylene-, gibberellin-, cytokinin-, and brassinosteroid-responsive genes showed a negative correlation with caterpillar-induced genes, genes that were highly induced within 30 min after hormonal treatment, and genes that moderately increased 3 h after hormonal treatment. A dendrogram analysis of the data showed that hormone-related gene expression gradually changed from 1 to 24 h, and that responses in the first hour after caterpillar feeding were distinct from those observed at later time points (Fig. 3). This suggests that major hormonal induction occurred within one hour after caterpillar infestation.

**Fig. 4.**
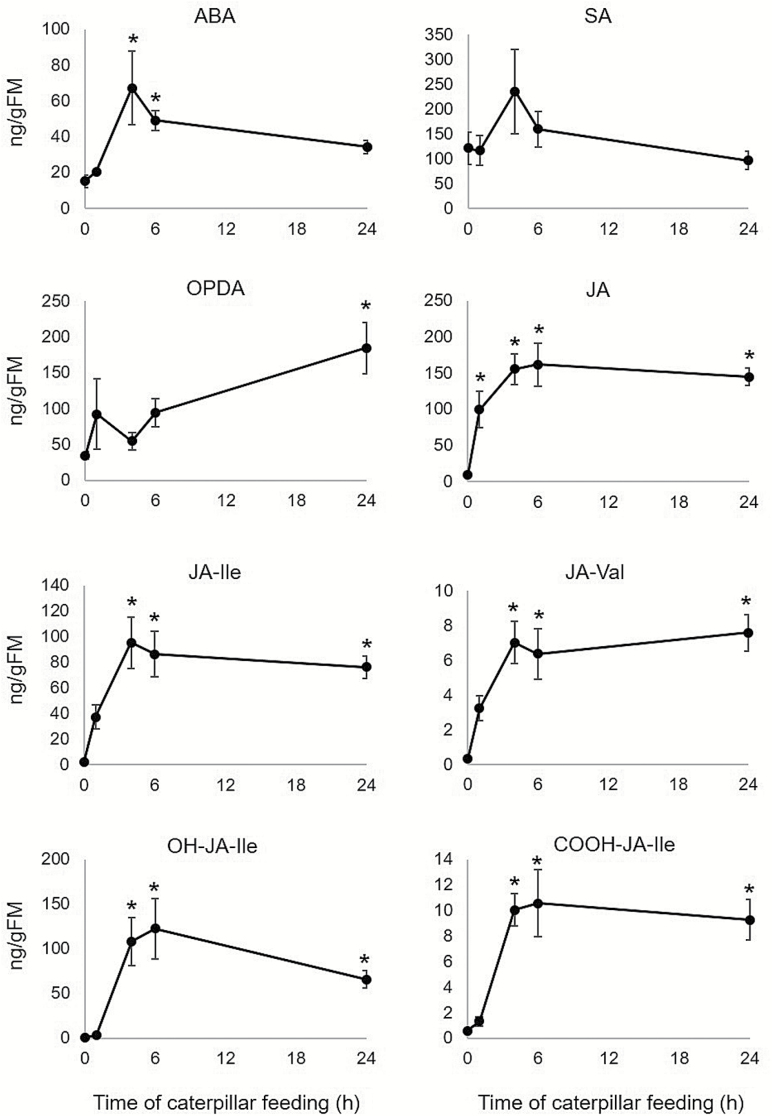
Plant phytohormones produced after *S. exigua* feeding on maize leaves. ABA, abscisic acid; JA, jasmonic acid; SA, salicylic acid. Mean±SE of *n*=5. *P<0.05, Student’s *t*-test relative to uninfested control.

We measured changes in phytohormone levels induced by *S. exigua* feeding using LC-MS/MS. As shown in Fig. 4, the ABA level increased significantly 4 and 6 h after the initiation of caterpillar feeding. Although SA levels showed a similar trend, the induction was not significant. The JA level increased significantly from 1 to 24 h, and JA conjugates (JA-Val, JA-Ile) were highly induced after 4–24 h of caterpillar feeding. Deactivation products of JA-Ile (OH-JA-Ile and COOH-JA-Ile) also increased in response to herbivory, suggesting that there was negative feedback regulation and attenuation of active jasmonate levels (Koo and Howe, 2012). The JA precursor OPDA increased in abundance only after 24 h of caterpillar feeding (Supplementary Table S3).

### Caterpillar-induced changes in jasmonic acid biosynthesis

**Fig. 5.**
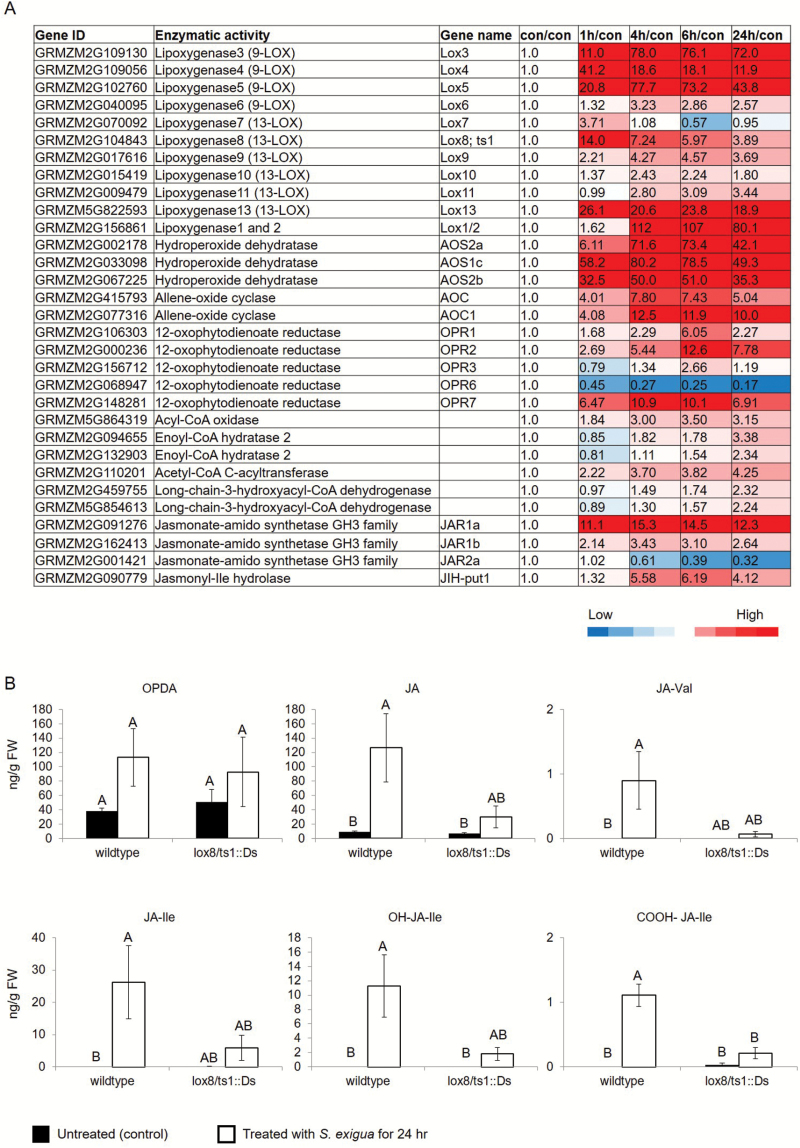
Effects of caterpillar feeding on jasmonic acid biosynthesis. (A) Heat map of gene expression that is related to jasmonic acid (JA) metabolism. Values are presented as fold change relative to untreated control samples. Mean±SE, *n*=4. (B) JA and JA conjugate levels in a *lox8/ts1::Ds* gene knockout line in response to caterpillar attack. Black bars, untreated; white bars, caterpillar infestation for 24 h. Different letters above the bars indicate significant differences, *P*<0.05, ANOVA followed by Tukey’s HSD test. (This figure is available in color at *JXB* online.)

Although JA, SA and ABA were all affected by *S. exigua* feeding (Figs 3 and 4), the greatest induction at both the gene expression and metabolite level involved JA and related metabolites. JA significantly increased after 4–24 h of caterpillar feeding and, in addition, the biosynthesis of jasmonoyl–amino acid conjugates was induced after 4 h of caterpillar feeding. Maize lipoxygenases (LOX) initiate fatty acid oxidation pathways for the synthesis of compounds that function in plant defense against insect herbivory (Porta and Rocha-Sosa, 2002; Christensen *et al.*, 2015) (Supplementary Fig. S6A). The up-regulation of gene expression (Supplementary Fig. S4), as well as the hormonal response signatures (Fig. 3) and phytohormone quantification (Fig. 4), suggested that caterpillar feeding elicited the production of a complex array of oxylipins. Therefore, we investigated the expression of genes associated with oxylipin and JA production (Dave and Graham, 2012; Koo and Howe, 2012; Borrego and Kolomiets, 2016) http://www.plantcyc.org;Fig. 5A) in more detail. In general, the first steps of the pathway are highly induced by caterpillar feeding. Lipoxygenases that enable the production of 12-oxo-phytodienoic acid (12-OPDA) and its downstream JA products (13-LOXs; *LOX7*, *9*, *10*, *11* and *13*) were induced from 4 to 24 h. Another 13-LOX gene, *LOX8/ts1* (GRMZM2G104843), was highly induced beginning in the first hour of infestation. A similar pathway involving 9-LOX activity on linolenic and linoleic acid (*LOX3*, *4*, *5* and *6*), which leads to the 12-OPDA positional isomer, 10-oxo-11-phytodienoic acid (10-OPDA) and 10-oxo-11-phytoenoic acid (10-OPEA), was highly induced. A comparative analysis of oxylipin biosynthetic genes demonstrated that 9-LOXs were induced to higher levels than 13-LOXs in response to herbivory (Fig. 5A). Comparable to the 13-LOX-derived linolenate oxidation that results in 12-OPDA and JA, 9-LOX-derived linoleic acid oxidation enables 10-oxo-11-phytoenoic acid production and a series of C14 and C12 cyclopentones, collectively termed death acids (DAs; Supplementary Fig. S6A; Borrego and Kolomiets, 2016). In maize, fungal infection by southern leaf blight (*Cochliobolus heterostrophus*) has been found to cause induction of 9-LOXs and production of 10-OPEA, displaying local phytoalexin activity (Christensen *et al.*, 2015). As 9-LOXs are also strongly induced in response to herbivory, we hypothesize that DAs may have a direct defense or signaling function in response to caterpillar feeding.

Allene oxide synthase (*AOS*) genes, which encode the second step of the jasmonic acid pathway, were also highly induced. In addition, allene-oxide cyclase (*AOC*) genes, which encode the third step in the pathway, were up-regulated at all time points. Elevated JA levels have been associated with insect resistance in several plant species (Ellis *et al.*, 2002; Shivaji *et al.*, 2010; Christensen *et al.*, 2013). The expression patterns of oxophytodienoate reductase genes (*OPR*), which encode the fourth step of the jasmonic acid pathway, were varied: *OPR7* was highly induced, whereas *OPR1* and *OPR2* only increased slightly after caterpillar feeding. Moreover, the expression levels of *OPR3* and *OPR6* decreased. Expression of the genes for the subsequent enzymatic steps, catalysed by acyl-CoA oxidase, enoyl-CoA hydratase, acetyl-CoA *C*-acyltransferase and long-chain-3-hydroxyacyl-CoA dehydrogenase, increased slightly after 4 h or more of caterpillar feeding (Fig. 5A). This suggests that other intermediates of the oxylipin pathway may also have functions in plant defense.

JA is conjugated to the amino acid isoleucine (Ile) by JAR1 (JASMONATE RESISTANT1; Supplementary Fig. S5A; Staswick *et al.*, 2002) to form JA-Ile, the major active form of the JA phytohormone in plants (Koo and Howe, 2012). The maize genome contains five *JAR1*-like isoforms that group into two gene clusters (Borrego and Kolomiets, 2016). In our transcriptomic dataset, two *JAR1* cluster genes, *JAR1a* (GRMZM2G091276) and *JAR1b* (GRMZM2G162413), were highly induced, whereas the expression of the *JAR2* cluster gene *JAR2a* (GRMZM2G001421) decreased (Fig. 5A). The other two *JAR2* genes, *JAR2b* (GRMZM2G060991) and *JAR2c* (GRMZM2G061005), were not detected in our dataset. Similar results have been described in rice (*Oryza sativa*), where two genes encoding JAR1-like-GH3 enzymes, *OsJAR1* and *OsJAR2*, showed different expression patterns in response to different stresses (Wakuta *et al.*, 2011). However, only OsJAR1 activity seems to be required during defense against the blast fungus *Magnaporthe grisea*, and the *osjar1* mutant also showed JA-related developmental modification (Riemann *et al.*, 2008). Changes in maize *JAR1* gene expression may indicate the function of these enzymes in forming JA–amino acid conjugates (Fig. 3). In Arabidopsis, the JAR1-GH3 enzyme family (adenylate-forming enzymes) conjugates amino acids to diverse acyl acids (Meesters *et al.*, 2014), including phytohormones JA, IAA and SA (Staswick *et al.*, 2002; Zeier, 2013; Westfall *et al.*, 2016; Urano *et al.*, 2017). Thus, this enzyme mediates crosstalk between auxin, developmental, and pathogen response pathways. However, it is not known whether the GH3 enzyme family mediates similar changes in maize because, to date, no JAR-like isoform has been functionally characterized in maize (Borrego and Kolomiets, 2016; Lyons *et al.*, 2013).

The jasmonoyl–amino acid conjugate JA-Ile is the most effective ligand for the SCF (COI1) receptor (Fonseca *et al.*, 2009). Furthermore, the catabolism of JA-Ile can be a mechanism to reduce JA effects on downstream signaling (Koo and Howe, 2012). In *N. attenuata*, the *NaJIH* gene, which encodes a jasmonyl-Ile hydrolase, has been associated with hydrolysis of JA-Ile (Woldemariam *et al.*, 2012). The putative maize JIH gene, GRMZM2G090779, was induced after 6 h of caterpillar feeding (Fig. 5A), similar to the increase in the likely downstream products from this reaction, OH-JA-Ile and COOH-JA-Ile (Fig. 4). Together, these results support the previous finding of a negative feedback regulation of jasmonic acid active form (Koo and Howe, 2012). So far, no experimental evidence linking the expression-level regulation of these genes to JA catabolism has been described (Lyons *et al.*, 2013; Borrego and Kolomiets, 2016).

As *LOX8/ts1* (13-LOX) expression was strongly induced in response to *S. exigua* feeding (Fig. 5A), we genetically investigated the role of this gene in caterpillar-induced jasmonate production using two different *LOX8/ts1* knockout alleles (*lox8/ts1::Ds* and *lox8/ts1-ref*). While *S. exigua* elicited significantly lower levels of several jasmonates in the *lox8/ts1::Ds* (Fig. 5B) and *lox8/ts1-ref* (Supplementary Fig. S6B) mutants, they were not completely devoid of 12-OPDA derivatives, suggesting that multiple 13-LOXs provided the substrate for jasmonate biosynthesis. These results are consistent with our expression data showing the herbivore-induced transcript accumulation of multiple 13-LOXs, which parallels previous findings in maize and Arabidopsis (Chauvin *et al.*, 2013; Christensen *et al.*, 2013). Interestingly, investigation of caterpillar growth demonstrated no difference between the *lox8* mutant and W22 wild-type plants (Supplementary Fig. S7). Similarly, a previous study in Arabidopsis showed that the *Atlox6* mutant had significantly reduced jasmonate production compared with wild-type plants, but differences in caterpillar growth were not observed (Chauvin *et al.*, 2013). The observation that *Lox8* knockout mutations do not fully abrogate defense against *S. exigua* suggests that there is redundancy in the function of maize 13-Lox genes. For instance, *Lox13*, which is also strongly induced by caterpillar feeding, could substitute for *Lox8* function.

### Benzoxazinoid biosynthesis is involved in herbivore defense mechanisms

**Fig. 6.**
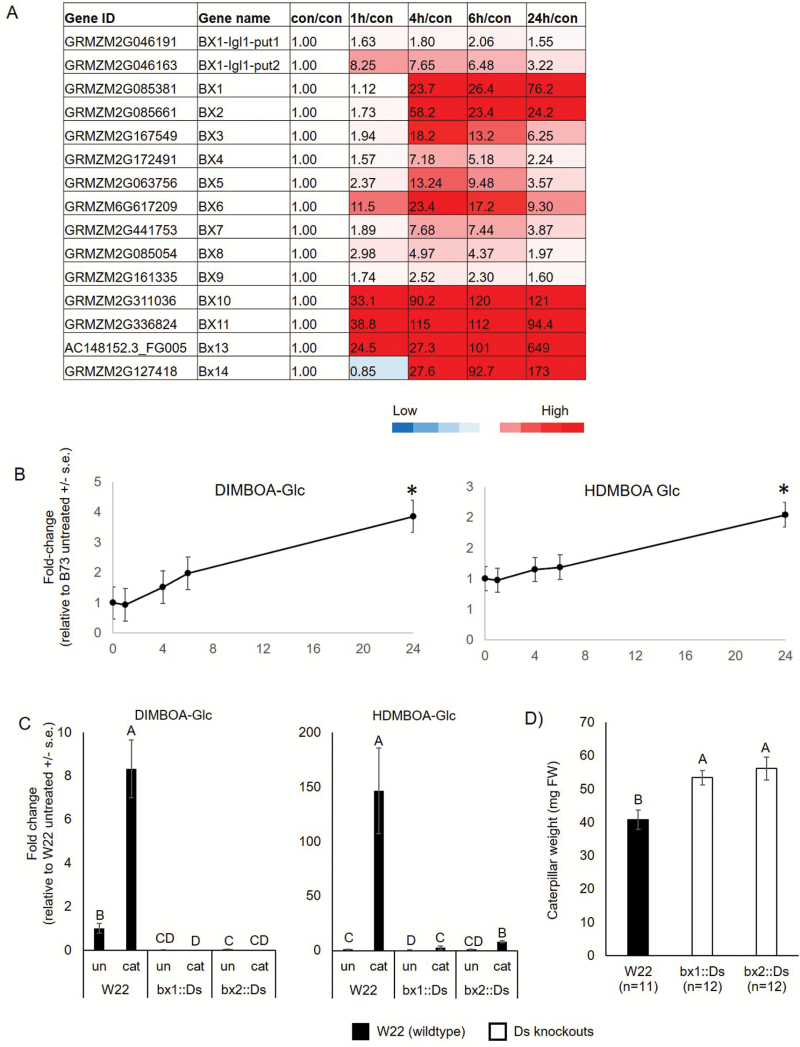
Effects of caterpillar feeding on benzoxazinoid-related genes and metabolites. (A) Heat map of gene expression and (B) DIMBOA-Glc and HDMBOA-Glc abundance over time after caterpillar feeding. Values are presented as fold change relative to untreated control. Mean±SE of *n*=4 for transcriptomic data, *n*=5 for metabolomic data). (C) Abundance of DIMBOA-Glc and HDMBOA-Glc in wild-type W22, *bx1::Ds*, and *bx2::Ds*, with and without caterpillar feeding. Different letters above the bars indicate significant differences, *P*<0.05, ANOVA followed by Tukey’s HSD test. (D) *S. exigua* caterpillar body weight after 4 d on wild-type W22, *bx1::Ds*, and *bx2::Ds* mutant plants. (This figure is available in color at *JXB* online.)

The role of benzoxazinoids in defense against herbivory has been studied extensively in maize (Frey *et al.*, 2009; Ahmad *et al.*, 2011; Adhikari *et al.*, 2015; Fig. 1). Therefore, we investigated benzoxazinoid gene expression and function in response to *S. exigua* feeding (Fig. 6A and Supplementary Table S13). As shown in Fig. 6A, *Bx1*, *Bx2*, *Bx3*, and *Bx6* transcripts were highly induced from 4 to 24 h, whereas *Bx4*, *Bx5*, *Bx8*, *Bx9*, and *Bx7* were highly induced after 4 and 6 h of caterpillar infestation. In contrast, *Bx10*, *Bx11*, and *Bx13* expression already increased after 1 h of caterpillar infestation, suggesting that an immediate response to caterpillar feeding is the conversion of DIMBOA-Glc to HDMBOA-Glc and/or DIM2BOA-Glc (Fig. 1). However, *Bx14*, which is required for HDM2BOA-Glc synthesis, was induced only after 4 and 6 h of caterpillar infestation. Both DIMBOA-Glc and HDMBOA-Glc abundances gradually increased from 4 to 24 h (Fig. 6B).

To further investigate the effect of *S. exigua* feeding on benzoxazinoid content in maize, we employed the previously identified *bx1::Ds* and *bx2::Ds* mutations in the W22 genetic background (Betsiashvili *et al.*, 2015; Tzin *et al.*, 2015*a*). DIMBOA-Glc and HDMBOA-Glc levels significantly increased in the W22 wild-type treated with caterpillars compared with the untreated plants (Fig. 6C). We observed a similar induction of these metabolites after caterpillar feeding in the B73 inbred line (Fig. 6B). In contrast, the levels of these compounds were very low in the W22 knockout lines, *bx1::Ds* and *bx2::Ds*, even after caterpillar infestation. At least two other maize genes, GRMZM2G046191 (*IGL1*) and GRMZM5G841619 (*TSA1*), encode the same indole-3-glycerol phosphate lyase activity as *Bx1* (Frey *et al.*, 2000; Kriechbaumer *et al.*, 2008). Thus, the absence of DIMBOA-Glc and HDMBOA-Glc induction by caterpillar feeding on the *bx1::Ds* mutant indicates either metabolic channeling or that the other two indole-3-glycerol phosphate lyases are not expressed. The latter hypothesis is supported by the fact that *IGL1* and *TSA1* expression is not strongly induced by caterpillar feeding (Supplementary Tables S7 and S13). Additionally, the clustering of maize gene expression patterns shows that *Bx1* is co-expressed with the other *Bx* genes, whereas *IGL1* and *TSA1* cluster with genes responsible for tryptophan and volatile indole biosynthesis, respectively (Wisecaver *et al.*, 2017). The abundance of HDMBOA-Glc significantly increased in *bx2::Ds* plants after caterpillar infestation. It is possible that the other cytochrome P450 enzymes in the pathway (Bx3, Bx4, or Bx5), or perhaps other maize cytochrome P450s, catalyse the initial Bx2 indole oxidation reaction to a more limited extent.

Corn leaf aphids (*R. maidis*) grow better on *bx1::Ds* and *bx2::Ds* mutant lines than on wild-type W22 (Betsiashvili *et al.*, 2015; Tzin *et al.*, 2015*a*). To determine whether this is also the case for *S. exigua*, caterpillar mass was measured after 4 d of feeding on mutant and wild-type seedlings. There was a significant increase in caterpillar body mass on *bx1::Ds* and *bx2::Ds* mutant seedlings relative to wild-type W22 (Fig. 6D). A similar increase in body weight was observed with *S. littoralis* feeding on *bx1* mutant plants relative to wild-type maize inbred line B73 (Maag *et al.*, 2016). This result confirms the function of benzoxazinoids as defensive molecules that affect caterpillar growth through direct toxicity or reduced growth due to aversive effects. It is unlikely that benzoxazinoid biosynthesis is the only defense mechanism that is induced by caterpillar feeding on maize plants. Other genes that have been previously implicated in plant defense are also induced. For instance, the induction of the cellulose and suberin biosynthesis pathways is an indication that cell walls are reinforced as a mechanical defense mechanism (Appel *et al.*, 2014; Schwachtje and Baldwin, 2008; Tzin *et al.*, 2015*a*). Similarly elevated phenylpropanoid biosynthesis (Table 1) can lead to both the production of defense-related metabolites and enhanced mechanical defenses against caterpillar feeding.

## Conclusion

In this study, we examined the dynamic effects of caterpillar feeding on maize, one of the world’s most important crop plants. Transcriptomic and metabolomic assays showed rapid responses in the first hour after the initiation of caterpillar feeding, and continued changes in both the transcriptome and metabolome up to and including the 24 h time point. Our integrative analysis of these datasets demonstrates the function of genes contributing to two major defense-related pathways, benzoxazinoid and jasmonic acid biosynthesis. Future research on benzoxazinoids and phytohormones induced by *S. exigua* feeding will enable the breeding of maize cultivars with enhanced resistance to lepidopteran herbivores. In addition, our large transcriptomic and metabolomic datasets can be further utilized to discover previously unknown genes and metabolites that function in maize responses to biotic stress.

## Supplementary data

Supplementary data are available at *JXB* online.

Fig. S1. Design of the caterpillar feeding experiments.

Fig. S2. Comparison of RNAseq and qRT-PCR gene expression data from two independent sets of experimental samples.

Fig. S3. Venn diagram describing the number of genes up- or down-regulated by caterpillar infestation after 1 h and 24 h relative to the control and 1 h relative to 24 h.

Fig. S4. *k*-Means clustering of genes expressed during caterpillar infestation. Gene expression in FPKM log2 values after *S. exigua* feeding over a 0–24 h time course.

Fig. S5. Effects of caterpillar feeding on phenylpropanoid biosynthesis. 

Fig. S6. Effects of caterpillar feeding on jasmonic acid pathway.

Fig. S7. *S. exigua* caterpillar body weight after 4 d on wild-type (*Lox8/ts1*), and homozygote mutant (*lox8/ts1::Ds*) plants. 

Table S1. Primers used for quantitative RT-PCR analysis.

Table S2. Parameters of benzoxazinoid metabolites detected by LC-MS/MS.

Table S3. Phytohormone analysis of B73 leaves in response to *S. exigua* feeding. 

Table S4. Primers used to screen for knockout mutations.

Table S5. RNAseq raw data.

Table S6. RNAseq raw data after data filtering (genes that had expression values of zero more than three times were excluded).

Table S7. RNAseq data for four caterpillar feeding time points after Cuffdiff.

Table S8. LC-MS/MS data from negative ion mode.

Table S9. LC-MS/MS data from positive ion mode.

Table S10. LC-TOF-MS data used for Fig. 2E from negative and positive ion modes.

Table S11. List of differentially expressed maize genes for at least one time point (±>2-fold changed) with *P* value<0.05, FDR adjusted, used for PCA analysis, clustering over-representation, and PageMan analysis.

Table S12. Orthologous Arabidopsis and maize genes used for Hormonometer analysis.

Table S13. RNAseq data of benzoxazinoid genes for four caterpillar feeding time points after Cuffdiff (v3.20).

## Supplementary Material

Supplementary Figures S1-S7

Supplementary Tables S1-S13

## Abbreviations

DIMBOA: 4-dihydroxy-7-methoxy-1,4-benzoxazin-3-one
FPKM: fragments per kilobase of exon per million fragments
Glc: glucoside
HDMBOA: 2-hydroxy-4,7-dimethoxy-1,4-benzoxazin-3-one
JA: jasmonic acid
SA: salicylic acid.
