# Supplementary material for: Rapid defense responses in maize leaves induced by Spodoptera exigua caterpillar feeding
Source: J Exp Bot. 2017 Aug 22;68(16):4709–23. doi: 10.1093/jxb/erx274 (PMC5853842; doi:10.1093/jxb/erx274)
Supplement: Supplementary Tables S1-S13 [file erx274_suppl_supplementary_tables_s1-s13.pdf]

Figure S1.

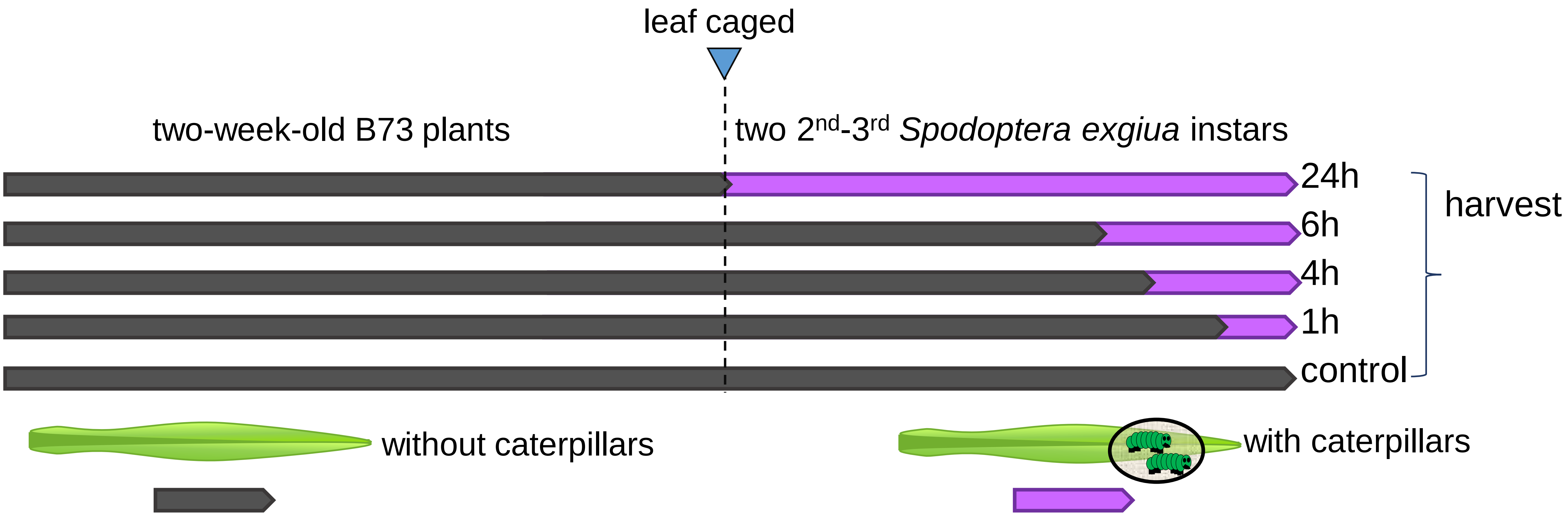

Supplementary Figure S1. Design of the caterpillar feeding experiments. The third leaf of two-week-old B73 maize plants was enclosed in a clip cage. At staggered intervals, two second to third instar *S. exigua* caterpillars were added to each cage. Leaf tissue was harvested after 1 hr to 24 hr of caterpillar feeding. All samples, including controls (untreated), were harvested within a 45-min time period. Harvested tissue was used for assays of gene expression by Illumina sequencing, as well as for metabolite profiling.

Figure S2.

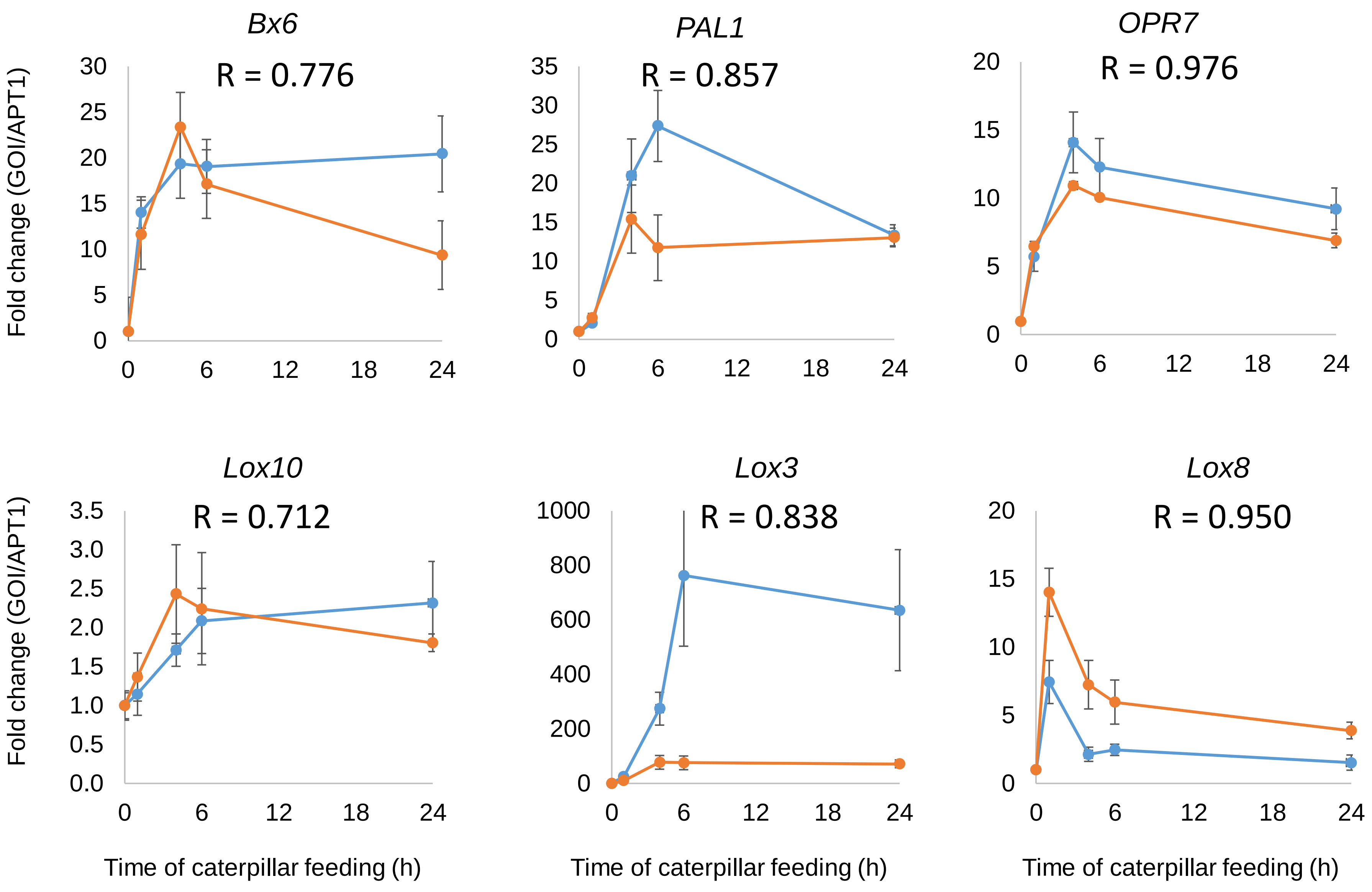

Supplementary Figure S2. Comparison of RNAseq and qRT-PCR gene expression data from two independent sets of experimental samples. The following genes were measured by qRT-PCR and RNAseq and normalized to Adenine Phosphate Transferase 1 (*APT1*): GRMZM2G131907 - its expression level did not change across the caterpillar infestation period, *Bx6* - GRMZM6G617209, Phe Ammonia-lyase (*PAL*) - GRMZM2G063917, 12-oxo-phytodienoic acid reductase 7 (*OPR7*) - GRMZM2G148281, Lipoxygenase 10 (*LOX10*) - GRMZM2G015419, Lipoxygenase 3 (*LOX3*) - GRMZM2G109130, and Lipoxygenase 8 (*LOX8*) - GRMZM2G104843. Mean +/- SE of n = 4, for RNAseq (orange) and n = 5 for qRT-PCR (blue). *R* value = correlation coefficient.

Figure S3.

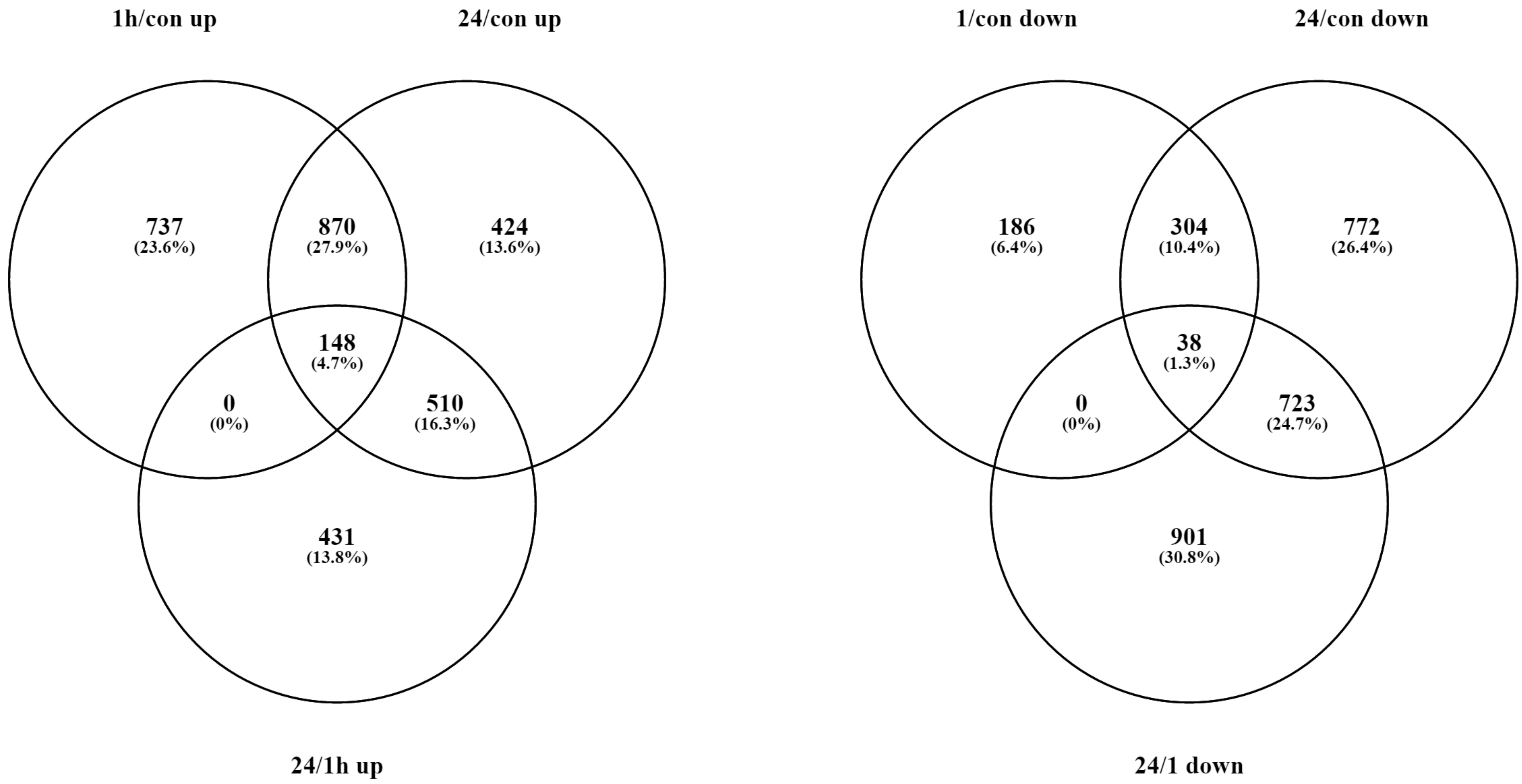

Supplementary Figure S3. Venn diagram describing the number of genes up- or down-regulated by caterpillar infestation after 1 hr and 24 hr relative to the control and 1 hr relative to 24 hr. *P* value < 0.05 FDR and fold change > 2 or < 0.5.

Figure S4.

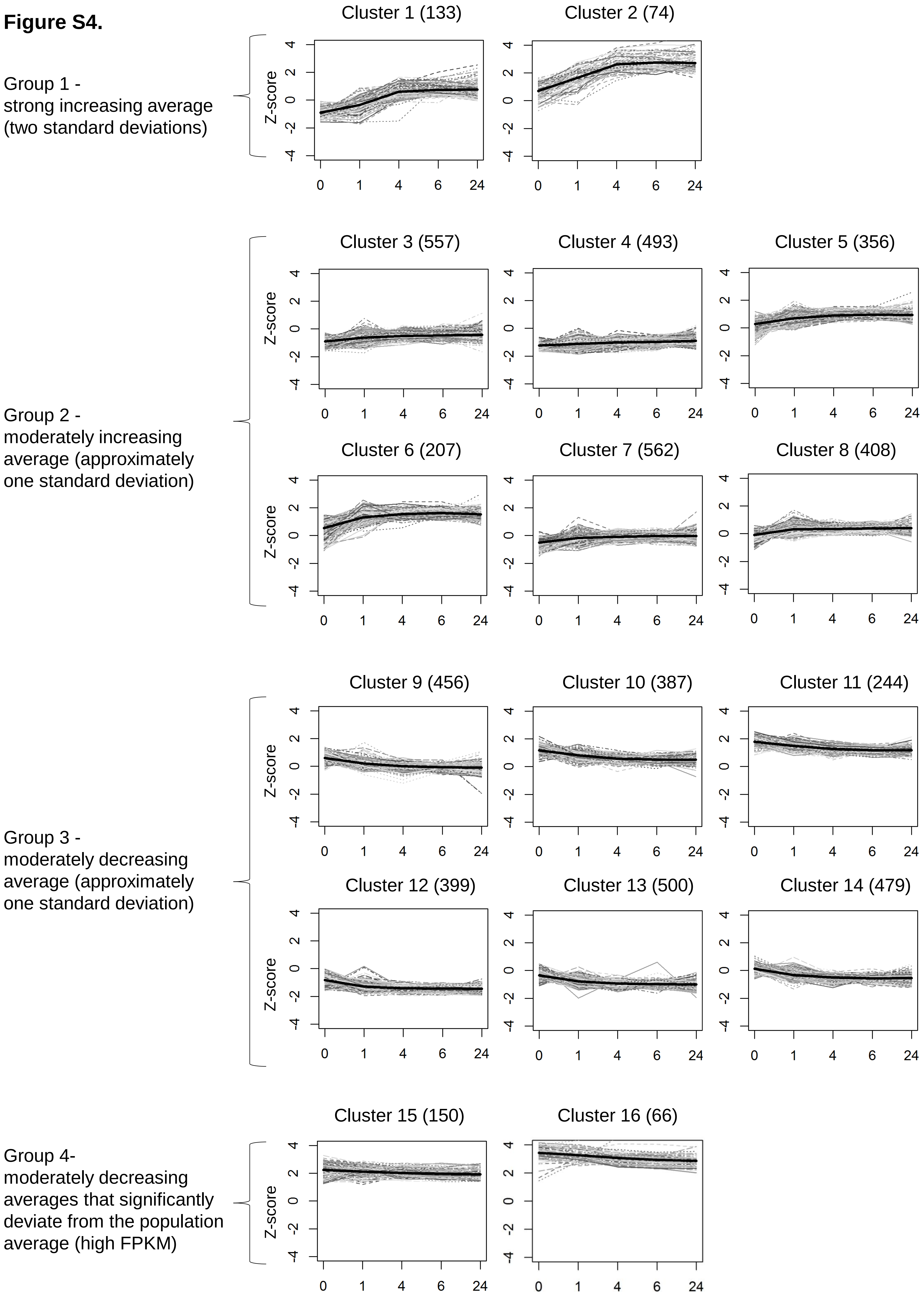

Supplementary Figure S4. *K*-means clustering of genes expressed during caterpillar infestation. Gene expression in FPKM log2 values after *S. exigua* feeding over a 0–24 hr time course. Genes were selected based on the following parameters: *P* value < 0.05 FDR and fold change > +/- 2 (5,470 genes). In bold: Z-score represents the number of standard deviations between the genes represented in the cluster and the mean of the population distribution. In brackets: the number of genes in each cluster.

Figure S5

A)

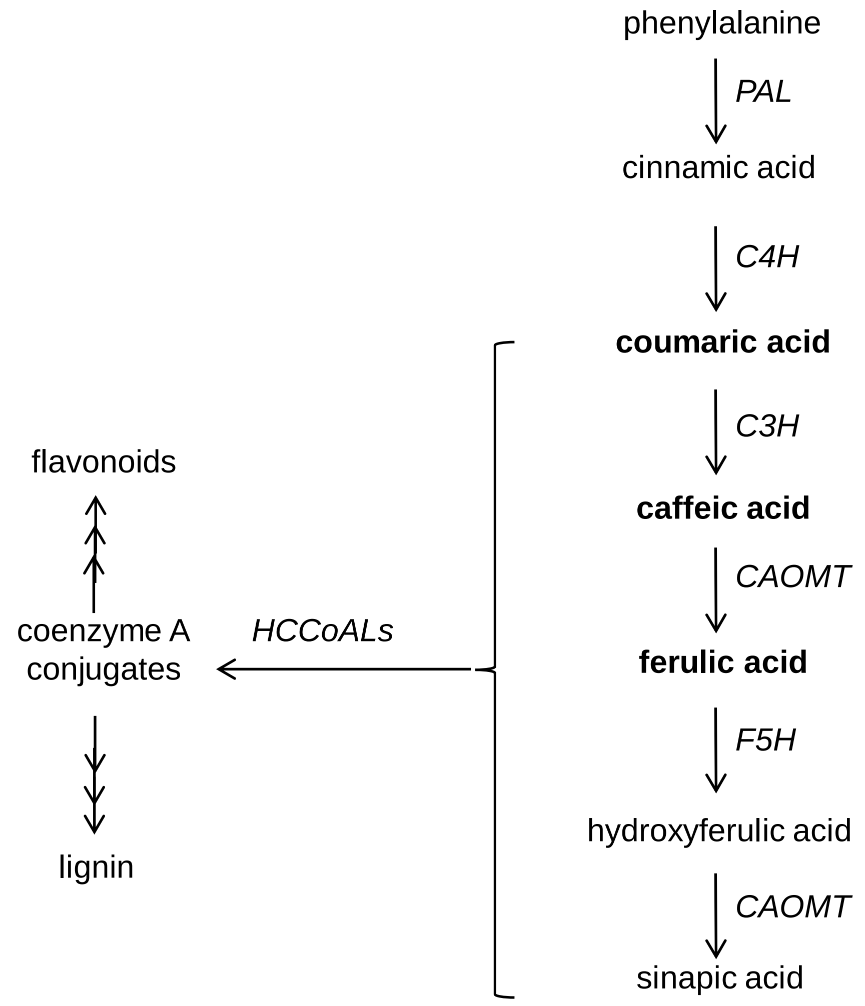

B)

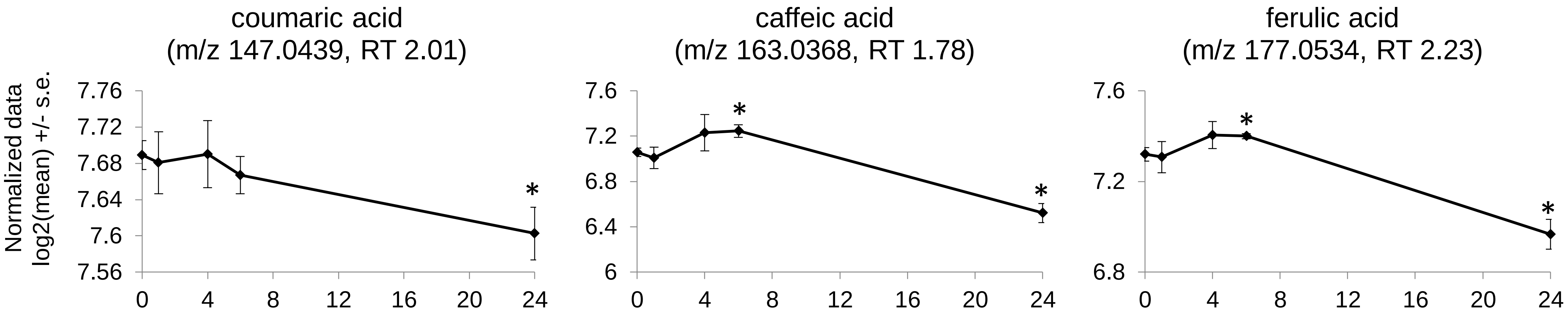

Supplementary Figure S5. Effects of caterpillar feeding on phenylpropanoid biosynthesis. A) A schema of the initial pathway of phenylpropanoid biosynthesis. In bold, metabolites that were identified in the LC-MS dataset. B) The normalized levels of three phenylpropanoid metabolites. The MS/MS spectrum was compared to the RIKEN MSn Spectrum Database for Phytochemicals (ReSpect; http://spectra.psc.riken.jp/). **P* < 0.05, Student's *t*-test. Mean +/- SE of n = 5.

Figure S6

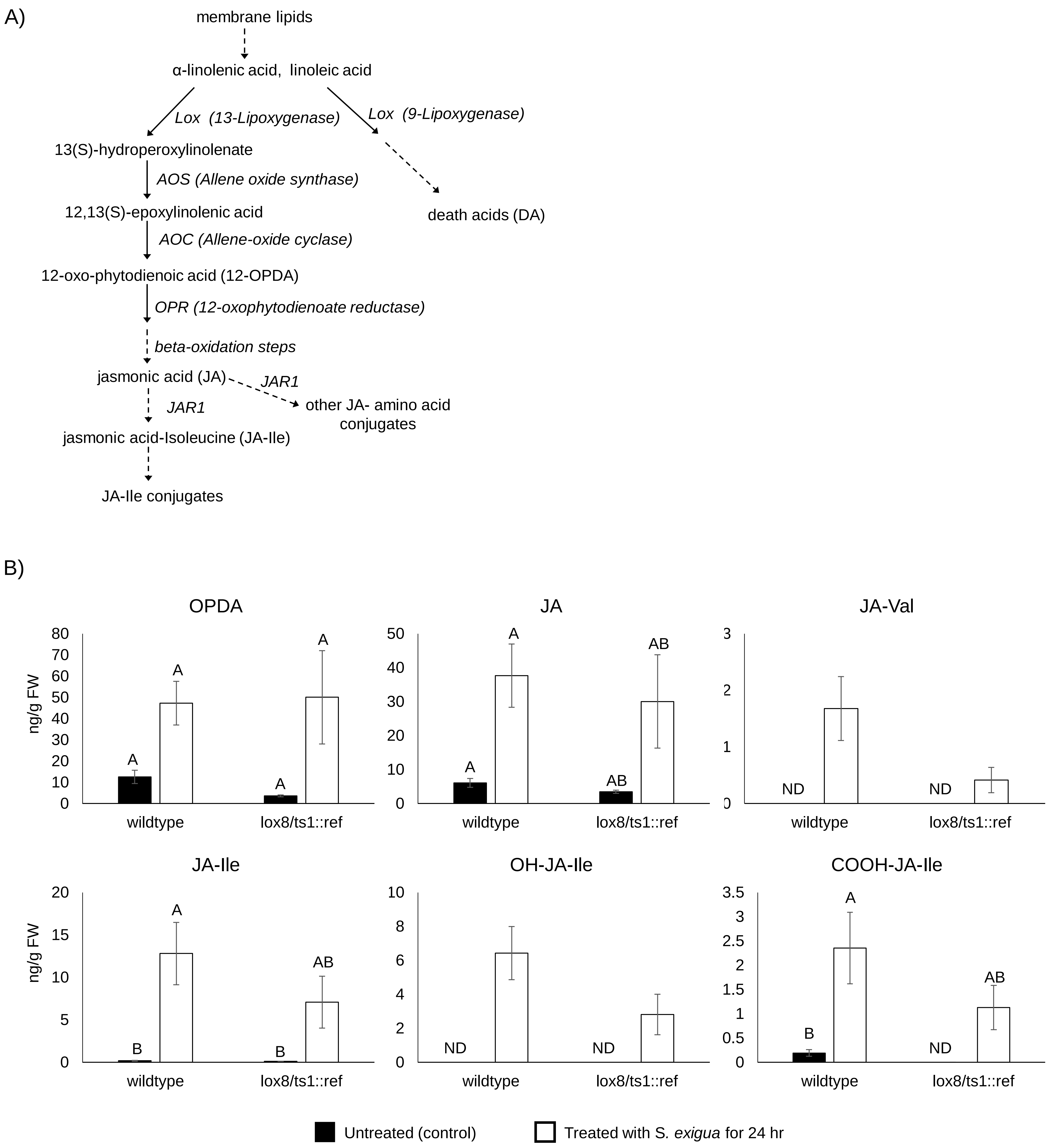

Supplementary Figure S6. Effects of caterpillar feeding on jasmonic acid pathway. A) The jasmonic acid biosynthesis and catabolic pathway in maize. B) JA and JA conjugate levels of *lox8/ts1-ref* (*LOX8*; GRMZM2G104843) gene knockout in response to caterpillar attack. Black bars: untreated, white bars: caterpillar infestation for 24 hr. Different letters above the bars indicate significant differences, $P < 0.05$, ANOVA followed by Tukey's HSD test. ND = not detected.

Figure S7

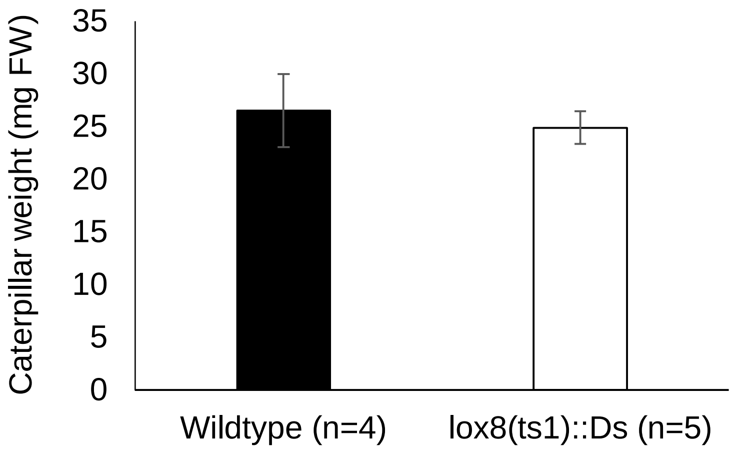

Supplementary Figure S7. *S. exigua* caterpillar body weight after four days on wildtype (*Lox8/ts1*), and homozygote mutant (*lox8/ts1::Ds)* plants. Mean +/- SE of n = 4-5. *$P < 0.05$ Student's *t*-test relative to uninfested control showed no significant differences between the plants.
